# Review: The Nutritional Management of Multiple Sclerosis With Propionate

**DOI:** 10.3389/fimmu.2021.676016

**Published:** 2021-07-28

**Authors:** Derek Tobin, Runar Vige, Philip C. Calder

**Affiliations:** ^1^DAHRT Biocare AS, Kolsås, Norway; ^2^School of Human Development and Health, Faculty of Medicine, University of Southampton, Southampton, United Kingdom; ^3^NIHR Southampton Biomedical Research Centre, University Hospital Southampton NHS Foundation Trust and University of Southampton, Southampton, United Kingdom

**Keywords:** short chain fatty acid, propionate, microbiota, immunity, auto-immune, multiple sclerosis

## Abstract

Over the last 15 years there has been an accumulation of data supporting the concept of a gut-brain axis whereby dysbiosis of the gut microbiota can impact neurological function. Such dysbiosis has been suggested as a possible environmental exposure triggering multiple sclerosis (MS). Dysbiosis has been consistently shown to result in a reduction in short-chain fatty acid (SCFA) producing bacteria and a reduction in stool and plasma levels of propionate has been shown for MS patients independent of disease stage and in different geographies. A wealth of evidence supports the action of propionate on T-cell activity, resulting in decreased T-helper cell 1 (Th1) and T-helper cell 17 (Th17) numbers/activity and increased regulatory T cell (Treg cell) numbers/activity and an overall anti-inflammatory profile. These different T-cell populations play various roles in the pathophysiology of MS. A recent clinical study in MS patients demonstrated that supplementation of propionate reduces the annual relapse rate and slows disease progression. This review discusses this data and the relevant mechanistic background and discusses whether taming of the overactive immune system in MS is likely to allow easier bacterial and viral infection.

## Introduction

Multiple sclerosis (MS) is a chronic, progressive autoimmune disease for which there is no current cure. Worldwide, approximately 2.8 million people have MS, making it the most common neurological auto-immune disease (1). The development of MS is considered to result from a combination of genetic and environmental factors including childhood obesity, smoking, low Vitamin D levels and geographical latitude distant from the equator (2–4). More recently, the constitution and activity of the intestinal flora (microbiota) has been suggested as one of the environmental triggers for the development of MS (3). Here we review the data supporting the role the microbiota and short-chain fatty acid (SCFA) metabolites, in particular propionate, play in the pathophysiology of MS. Additionally, we discuss the conflicting goals of immunosuppression and the need to maintain an appropriate immune response to pathogenic bacteria and viruses.

## MS and the Gut-Brain Axis

Evidence of the gross pathophysiological effect of the gut content that may apply to MS has been demonstrated through fecal transplant studies. Using a murine model of MS [experimental autoimmune encephalomyelitis (EAE)], it was shown that MS score (based on motor deficits) worsened when mice received fecal material from patients with MS compared to mice receiving fecal material from healthy individuals (5). This study was repeated using twin donors where one was healthy and the other with MS. Fecal transplants resulted in a higher frequency of spontaneous EAE with the MS-human donor compared to that from the healthy human donor (6).

A major aspect of microbe–host communication receiving increased attention is the two-way communication between the gut microbiota and the central nervous system (CNS), the so-called gut–brain axis (7, 8). Gut-to-brain communication can occur *via* metabolite effects on the blood-brain-barrier (BBB), entero-endocrine factors, and systemic immune effects of microbe-derived metabolites such as SCFAs, products of tryptophan metabolism, phytoestrogens and bile acid metabolites (9).

A host of publications have described dysbiosis of gut microbiota in patients with MS and other autoimmune diseases compared to healthy controls (5, 6, 10–14). Interestingly, a common finding in these reports is that the alpha diversity (the variance within an individual) and beta diversity (variance between individuals and cohorts) are unchanged with MS. Rather, the dysbiosis is instead manifested as changes in the number of bacteria within the particular family or taxa. **Table 1** summarizes dysbiosis associated with development of MS.

**Table 1:** Comparison of the nature of dysbiosis reported in different studies in patients with MS.

Country	Subjects (n = those with MS)	Bacteria increased in MS	Bacteria decreased in MS	Reference
USA	RRMS n=31	Pseudomonas, Mycoplana, Haemophilus, Blautia, Dorea, Pedobacter, Flavobacterium	Prevotella, Parabacteroides, Adlercreutzia, Collinsella, Lactobacillus, Coprobacillus, Haemophilius	(11)
USA	RRMS n=60	Methanobrevibacter, Akkermansia	Butyricimonas, Prevotella	(12)
USA	RRMS n= 43	Ruminococcus	Fecalibacterium	(10)
USA	RRMS n=7	Akkermansia, Acinetobacter		(5)
Germany	MS twins n=34 (each twin pair had 1 with MS and one healthy)	Akkermansia		(6)
UK	RRMS n=39	Prevotella copri		(13)
Japan	RRMS n=20	Bifidobacteria, Streptococcus, Thermophilus, Eggerthella lenta	Bacteroides, Fecalibacterium, Prevotella, Anaerostipes, Clostridium, Sutterella	(14)
China	RRMS n=34	Streptococcus	Prevotella	(15)
Germany	RRMS and SPMS	Akkermansia, Faecalibacteria	Butyricimonas, Bacteroides, Romboutsia	(16)
Japan	RRMS n=62, SPMS n=15, Atypical MS n= 21, Controls n=55	RRMS: Bifidobacteria, Streptococcus.	RRMS: Megamonas	(17)
		SPMS: Streptococcus	SPMS: Roseburia	

Adapted from (15–17).

One unifying finding throughout studies reporting dysbiosis in patients with MS is the reduction in the number of SCFA-producing bacteria (5, 6, 10–12), as reviewed elsewhere (15). This is important because it shows that there are common findings on a functional level across studies, despite different microbiota profiles presumably due to background genetics, environmental differences and different nutritional habits. The reduction in SCFA producing bacteria in patients with MS, as discussed later, can have an important physiological impact.

## Propionate Deficiency in MS Patients

Five studies show that patients with MS have low levels of propionate in both feces and plasma (15–19). All 5 studies show that propionate levels in both feces and plasma are lower than those of healthy controls but there is less certainty for the other SCFAs, acetate and butyrate. Zeng et al. reported reduced fecal levels of all SCFAs in Chinese patients with MS compared to healthy controls (15). Interestingly the microbiota profile and level of SCFAs in feces were not affected by dietary and health habits (e.g., vegetarianism, physical activity, smoking, and alcohol intake), indicating that this pattern of dysbiosis may be a result of MS itself.

Park et al. assessed plasma SCFA levels in US patients with chronic MS (secondary progressive disease) and found significant reductions in acetate, propionate and butyrate (18). Duscha et al. measured SCFA levels in German patients with relapsing remitting MS (RRMS) and secondary progressive MS (SPMS) and found decreased propionate in plasma and feces for both MS subtypes, but no differences in butyrate and acetate (16). The findings between the two studies may be discordant with regards to acetate and butyrate levels due to the differences in the MS subtypes studied. However, both studies support a deficiency in propionate and a sub-analysis by Duscha et al. confirms that a propionate deficiency exists in both RRMS and SPMS.

Takewaki et al. studied 12 patients with RRMS and 9 patients with SPMS and showed reduced acetate, propionate and butyrate in the feces of RRMS patients, and a non-statistically significant reduction in SPMS patients (17). **Table 2** presents the outcomes of SCFA measurements in MS studies.

**Table 2:** SCFAs in patients with MS.

Author	Acetate		Propionate		Butyrate	
	Feces	Blood	Feces	Blood	Feces	Blood
(16) All types MS	No change from healthy controls	No change from healthy controls	Reduction p=0.0045	Reduction p=0.0006	No change from healthy controls	No change from healthy controls
(15) MS	Reduction	NA	Reduction	NA	Reduction	NA
	p=0.0001		p=0.0001		p=0.05	
(18) Patients with SPMS	NA	Reduction	NA	Reduction	NA	Reduction p=0.0001
		p=0.001		p=0.01		
(17) Patients with RRMS and SPMS	RRMS: Reduction p<0.001	NA	RRMS: Reduction p<0.001	NA	RRMS: Reduction p<0.001 SPMS:	NA
	SPMS: Reduction in levels but without statistical significance		SPMS: Reduction in levels but without statistical significance		Reduction in levels but without statistical significance	
(19) Clinically Isolated Syndrome, MS and healthy controls	NA	No difference between MS and healthy control	NA	Reduction reported for CIS/MS group. p=0.0008	NA	No difference between MS and healthy control
(20) RRMS and Clinically Isolated Syndrome	NA	Small but significant reduction in MS	NA	No difference between MS and healthy control	NA	No difference between MS and healthy control

Recently, Trend et al. have demonstrated a small but statistically significant reduction in propionate amongst patients with MS, without reductions in butyrate and acetate (19).

Contradictory data has come from the recent study of 58 patients with MS (a mix of RRMS and clinically isolated syndrome) and 50 healthy controls. Here, the serum level of acetate was significantly lower in MS patients but propionate and butyrate levels were similar in patients with MS and healthy controls (20).

Across the studies, there is a clear reduction in propionate levels in feces and plasma in patients with MS, independent of the subtype of MS and across different populations. These studies therefore provide complementary and consistent evidence that patients with MS have a dysbiosis leading to reduced numbers of SCFA producing bacteria which results in reduced levels of propionate across different geographies and disease forms.

## Mechanism of Immune Regulation by Propionate

As well as providing an energy source, SCFAs such as propionate and butyrate exert effects *via* 2 major mechanisms: 1) G-protein coupled receptors (GPRs) of the SCFA receptor family namely Free Fatty Acid Receptor 2 [FFA2 (formally known as GPR43)] and FFA3 (GPR41) (21, 22), and 2) histone deacetylase inhibition (HDACi) (23). A variety of immune cells express FFA2 and FFA3 as well as GPR109a for which butyrate is one of the proposed endogenous ligands. In contrast, T-cells lack the respective GPRs for mediating SCFA effects and therefore any direct modulation of T-cells by SCFAs is likely mediated by histone deacetylase inhibition (24).

An overview of the transporters and receptors for SCFAs and their distribution is presented in **Table 3**.

**Table 3:** SCFA transporters and receptors and tissue distribution in humans.

	Ligand	Tissue
**Transporters**		
MCT-1	Butyrate, lactate, pyruvate	Colon, Blood cells (monocytes, granulocytes, lymphocytes)
SMCT-1	Butyrate>propionate>acetate	Intestine (Ileum, proximal colon and distal colon)
**G-coupled protein receptors**		
FFA3 (GPR41)	Propionate=butyrate>acetate	Adipose tissue
		Peripheral blood mononuclear cells (PBMCs)
		Pancreas
		Spleen
		Placenta
		Monocytes, neutrophils, monocyte-derived dendritic cells (DCs)
FFA2 (GPR43)	Acetate=propionate=butyrate	Intestinal epithelium
		Monocytes, neutrophils, PBMCs, T and B cells
		Treg cells (colonic>spleen and mesenteric lymph node), colonic myeloid cells
GPR109a	Butyrate	Adipose tissue
		Colon
		Monocytes and macrophages

Ref (25, 26).

FFA2 is expressed on myeloid cells and some granulocytes. SCFAs act *via* FFA2 to induce the chemotaxis of neutrophils (27, 28) and neutrophil degranulation (29, 30).

Lipopolysaccharide (LPS) activated neutrophils showed diminished production of nitric oxide and TNF-α when co-cultured with propionate and both histone deacetylase and NF-kappa B activation were inhibited, suggesting their role in propionate-mediated inhibition of inflammation (31).

## Immune Regulation by SCFAs

The gut microbiota consists of bacteria, fungi and viruses. The mass of these micro-organisms in an adult is typically around 2 kg and could potentially evoke a debilitating and life-threatening immune response if left unchecked. In order to maintain a tolerogenic immune response in the gut, in the face of this considerable microbial load, communication between the commensal microbial population and the body’s immune system is essential to maintain immunological homeostasis (8, 9). T-cell maturation in the intestinal tract occurs in the gut associated lymphoid tissue (GALT) and from there cells migrate to the intraepithelial layer or lamina propria. These T-cells are highly modifiable and can be induced to develop into Treg, Th1, Th2, or Th17 cells. These modifications are regulated by metabolites such as SCFAs but also by interaction with antigen presenting cells [e.g., dendritic cells (DCs)] and intestinal epithelial cells (32). As discussed later, Treg, and Th17 cells are immune cells that play a central role in several auto-immune diseases and normalization of their activity may represent an important target for controlling MS.

Nutritional components and microbial metabolites such as acetate, propionate, butyrate, tryptophan and phytoestrogens, act as immune regulators. Numerous studies demonstrate the action of propionate in regulating T-cell activity *in vitro* (33–35), in animal *in vivo* studies (31, 33, 34, 36), and in human studies (16, 37–39). Supplementation with propionate enhances the activity and numbers of the anti-inflammatory Treg FoxP3^+^ cells and reduces the activity and numbers of Th17 and to a lesser extent Th1 pro-inflammatory T-cells *via* histone deacetylase inhibition (25, 34, 40–42).

Dendritic cells are important in the activation of T-cells. Human primary DCs express FFA3 and GPR109a but only small amounts of FFA2, thus allowing for regulation by SCFAs. *In vitro* analysis showed that both propionate and butyrate (but not acetate) reduced the DC expression of IL-6, and LPS-induced IL-12 and IL-23 (43). This would have a crucial role in reducing pro-inflammatory Th1 and Th17 populations and allow a shift to anti-inflammatory Treg cells. Additionally, the authors demonstrate SCFA specific effects on gene and protein expression of chemokines. Incubation of colon cultures from colitic mice with 1 mM SCFAs (representing gut levels) led to a reduction in pro-inflammatory chemokines with both propionate and butyrate but not acetate. Propionate reduced the expression of chemokine CC ligands (CCL3, CCL5 and CXCL9, CXCL10, CXCL11) representing an additional indirect effect of propionate towards infiltration of immune cells.

Propionate also has a direct effect on the inflammatory activity of non-immune cells shown by *in vitro* studies. Following LPS stimulation, NF-kappa B activity and TNF-α release were reduced when colon cultures were incubated with propionate or butyrate (44).

## Modification of the Immune System Through SCFAs Can Improve Auto-Immune Disease

There is evidence that regulation of T-cells by SCFAs and particularly butyrate has a beneficial effect on Parkinson's Disease (PD). Dysbiosis and reduced levels of butyrate and propionate have been demonstrated in patients with PD (45, 46). Supplemental butyrate reduced the alpha-synuclein deposition in gut nerve cells (enteroendocrine cells) (47) and clinical studies are underway to determine if SCFAs play a role in reversing the pathology of PD (48).

Intestinal inflammation such as in ulcerative colitis has been a target condition for microbiota and SCFA research. Studies to date mostly describe associations between the disease state, the immune inflammatory signature, dysbiosis and reduction in SCFAs (49). Propionate reduced IL-1, IL-6 and iNOS production in an *in vitro* model of ulcerative colitis (44) and administration of propionate during a 3-week period reduced intestinal inflammation in an animal model (34). In an animal model of colitis, colonic SCFA levels were associated positively with Treg activity and inversely with disease state (33), and supplemental propionate has been shown to regulate colonic motility (50), and intestinal inflammation in animal models of Irritable Bowel Disease (51). 

In patients with ulcerative colitis, butyrate inhibited the pro-inflammatory transcription factor NF-kappa B in macrophages and improved disease state as measured by the Disease Activity Index (52).

Propionate and other SCFAs have been investigated in relation to a number of other auto-immune disease states. Animal and *in vitro* studies have reported an association of propionate with metabolism, diabetes and hepatic steatosis (53–63), inflammation (35, 64–69) and colitis inflammation (70).

## The Effect of Propionate in Animal Models of Multiple Sclerosis 

The EAE animal model is often used for studying certain pathophysiological aspects relevant to MS. With this model, orally supplemented propionate was shown to ameliorate disease progression as measured by clinical scoring based on muscular function (e.g., tail tonicity, partial or total limb paralysis, death) (36, 71, 72). The studies consistently reported an associated increase in Treg cells and a decrease in Th17 cells with propionate supplementation. Of particular interest are the results from Haghikia et al. (36) demonstrating that feeding with propionate led to increased Treg cell frequency associated with the small intestine; transplantation of these cells in the EAE model improved the clinical outcome of the mice showing that gut associated T-cell responses were able to have systemic effects.

## Human Intervention Studies

In 2020, Duscha et al. confirmed that dysbiosis occurred in patients with MS and that levels of propionate, but not butyrate, were lower in MS patients compared to healthy controls (16). Previous work by the same group had shown propionate to be unique amongst SCFAs in its ability to improve disease score in the EAE animal model (36).

The study went on to investigate daily oral propionate supplementation in patients with MS. Patients showed significantly lower levels of Treg cells and significantly higher levels of Th17 cells at baseline compared to healthy controls representing a pro-inflammatory state for patients with MS. After 14 days of daily 1 g propionate supplementation, a significant reduction in Th17 cells and a significant increase in number and activity of Treg cells was seen.

Long term supplementation with 1 g propionate daily was performed in 97 patients with MS (in which patients had at least 1-year supplementation). Propionate intake was associated with a significant benefit to MS patients as measured by annual relapse rate and expanded disability status scale (EDSS) score.

The study had some design weaknesses including a non-treated control group rather than use of a blinded randomized placebo-controlled design. It is also unclear what medications were used for the treatment group *vs* the control group and whether medications changed during the 3 years of propionate supplementation. Despite these weaknesses, the data point to a beneficial effect of propionate supplementation in patients with MS.

## Safety of Propionate From an Immunological Perspective

Inflammation is a key mechanism in the defense of the body against invading pathogens. Many diets and nutrients are claimed to have anti-inflammatory properties with the presumption that this is always good. However, there could be concern that an anti-inflammatory agent could suppress inflammation needed during infection. At the same time, an uncontrolled inflammatory response is associated with chronic diseases such as diabetes, rheumatoid arthritis, allergies and auto-immune diseases. The question then is whether immune suppression leads to an open door to bacteria and viruses (73).

Interestingly, Kim et al. (74) showed propionate elicits complex immune regulation dependent on the immunological setting. The group demonstrated that *in vitro*, propionate mediated an increase in IL-10 production by T-cells in line with its anti-inflammatory properties, and propionate has been shown by others to be a central regulator of IL-10 production (16, 75). However, they also found that propionate-induced FoxP3^+^ expression (a marker of T reg cells) was dependent on the strength of T-cell activation. In conditions of high T-cell activation, SCFAs could suppress FoxP3^+^ cell induction whereas at low T-cell activity SCFAs enhanced FoxP3^+^ cell induction (from TGFβ1). This suggests that T-cell modulation by propionate and other SCFAs is in accordance with the immunological setting the T-cell is in. The group also suggest that SCFAs could facilitate the differentiation of T-cells to Th1 and Th17 cells given the right immunological conditions. The authors conclude that propionate “aids to promote the right type of T-cells for specific immunological conditions”.

The concept of appropriate T-cell modulation is further supported by detailed work by Bhaskaran et al. (76) looking at propionate effects in the light of mucosal infection, where an immune response is desired to fight infection but overt inflammation can lead to tissue damage. This is a common pathophysiological situation where initial inflammation is triggered in response to a pathogen to combat infection, but continued inflammation results in tissue damage. In the case of the study by Bhaskaran et al., propionate (and other SCFAs) again increased T reg cells, but during *Candida albicans* infection propionate also stimulated levels of Th17 cells and IL-17 and promoted clearance of the infection. These results in mice showed propionate improved the immune response against the mucosal fungal infection and at the same time promoted resolution of inflammation. Supporting *in vitro* data showed that a direct effect of propionate on Th17 cells led to reduced disease activity; however, co-culture with spleen and lymph node cells in a Th17 activated medium led to a switch in propionate activity and a promotion of IL-17 production.

Another example is given by the equine herpesvirus which enters the horse through the upper respiratory tract, is spread through infection of leukocytes and T-cells and causes neurological and reproductive disorders. Propionate (and other SCFAs) were shown to have a beneficial impact on the pathogenesis of the virus through several mechanisms including reduction in viral spreading through FFA2- and FFA3-mediated mechanisms (77). Viral spread to endothelial cells from monocytes was inhibited *via* an NF-kappa-B dependent pathway and inhibition of adhesion molecule expression. This mechanism may also be active in suppressing the spread of measles and herpes simplex virus (77).

A neutral effect of propionate was demonstrated with studies of the cholera vaccine. Cholera is an acute diarrheal disease resulting from bacterial infection. The cholera vaccine uses inactive (killed), whole bacteria that interact with antigen-presenting-cells and lymphocytes in the gut lymphoid tissue. Results from the study suggest that propionate and acetate have no detrimental effect on the response to the vaccine, whereas butyrate may even have beneficial effects (78).

Some contradictory results however point towards SCFAs having a detrimental effect during an infection. A study designed to specifically investigate the role of SCFAs in bacteria-induced inflammation was performed by Correa et al. They investigated the activity of neutrophils against a bacterial skin infection in an animal model. They showed that SCFAs had no effect on leukocyte accumulation but did reduce cytokine production and neutrophil phagocytic capacity suggesting a detrimental effect of SCFAs (79).

The relevance of the findings by Correa et al. can be seen in the light of a similar study by Ciarlo et al. (80) where morbidity and mortality were also measured in mice. Ciarlo et al., in agreement with Correa et al., demonstrated that propionate led to reduced activity of the innate immune system when mouse or human cells were challenged with a range of microbes (*Staph. aureus*, *Strep. pneumoniae*, *E. coli*, *Klebsiella pneumoniae*, *Candida albicans*). In this study, the production of inflammatory cytokines such as IL-6 and IL-12 (but less so for TNF-α) was reduced by propionate in macrophages and monocytes and to a lesser extent in DCs. Despite these effects, 3-week supplementation of infected mice with propionate had no effect on morbidity or mortality. Furthermore, despite the expected increase in Treg FoxP3^+^ cells following propionate treatment, the immunizing effect of a primary infection to subsequent infections from the same bacteria was not altered. The authors conclude that this was a successful demonstration that anti-inflammatory benefits associated with supplemental propionate did not come at a cost of depleted immune defense to pathogens and therefore supported the use of supplemental propionate.

Thus, the available data support a complex interplay between SCFAs and the immune system, whereby SCFAs in general, and propionate in particular, have a direct effect on T cell activity mediated by histone deacetylase inhibition which can be switched according to immunological context (e.g., chronic inflammation versus an infection).

The aim of this review is to consider the use of propionate in patients with MS in the light of the need for a fully functioning immune system. In the case of MS, an immune system running wild needs to be tamed, but not to the degree that pathogens cannot be controlled. The data collected to date suggest that supplemental propionate can promote a non-inflammatory T-cell profile leading to improved clinical outcomes for MS patients and that this occurs without compromising the immune response to pathogens.

## Dietary Management of MS With Propionate 

Although pharmacological treatment of MS has progressed significantly over the last decade or so, the is a continuing need for improved or novel ways of managing the disease. Most patients with MS still experience significant disease progression over time. The emerging importance of gut health and the microbiota in MS etiology offers an opportunity for new adjunctive tools in MS management.

Propionate is classified as a food product in the European Union (81) and the United States (21 CFR 184.1784) and is therefore considered safe for the general public. In the clinical study by Duscha et al. no serious adverse events were reported, and mild gastrointestinal adverse events were reported in less than 5% of participants.

Propionate is included in some food stuffs such as some breads and dairy products for enhancing shelf-life, but quantities are not included in product labels and a consumer would be unable to make informed dietary changes in order to control propionate intake. Therefore, the management of patients can only be through supplementation for which some examples now exist.

Gold et al. (82) reported dietary supplementation with 1g propionate daily as adequate for restoring plasma propionate concentration and immunological parameters in patients with MS to those of healthy individuals. Potential nutritional management of MS patients is described by Duscha et al. (16): 2 x 0.5 g capsules were given daily as an adjunct to disease modifying therapy. Participants under all MS drug regimens studied showed an increase in Treg cell numbers and function (except for where the drug glatiramer acetate was being used). Improved annual relapse rates were noted for all treatment groups (including the non-medicated), although participant numbers were low and results should be considered with caution.

How MS disease modifying treatments might affect the level of propionate or its actions has, to the best of our knowledge, not been investigated. The best indication of treatment effects on propionate levels comes from information on effects on microbiota.

Overall, few studies have been performed assessing the effect of MS-drugs on the microbiota, with inadequate data to draw conclusions. However, results to date suggest some medications may work to aid the levels of propionate. Dimethyl fumarate is an immune modifying treatment in MS with known side effects on gut health. A recent pilot study demonstrated dysbiosis in patients with MS compared with healthy controls and no significant differences were seen from dimethyl fumarate treatment apart from a trend towards increasing propionate (and butyrate) producing Bifidobacteria (83).

Microbiota profiling was performed in glatiramer acetate treated MS, dimethyl fumarate treated MS and healthy subjects (84) showing a tendency of dimethyl fumarate to enhance numbers of some SCFA producing bacteria. In a pilot study, glatiramer acetate treatment reduced the number of propionate-forming bacteria in MS patients whilst addition of Vitamin D somewhat restored these bacteria (10).

Given the safety profile of propionate, the potential clinical benefit in MS and its relatively inexpensive production, it can be considered that propionate is a good candidate agent for the dietary management of MS.

Thus, the current data suggests that patients with MS, either RRMS or SPMS, may have benefits from taking 1g propionate daily as an adjunct to their normal therapy. Studies of MS patients with propionate have been performed with patients on Interferon Beta, teriflunomide, glatiramer acetate, fingolimod, rituximab, and dimethyl fumarate. The numbers of participants studied by treatment regimen are low and the findings should be considered with caution. Adverse events are infrequent but may include gastrointestinal events.

## Conclusion

The role of propionate in MS is described as a story of dysbiosis, reduction of SCFA producing bacteria, reduced levels of plasma propionate coupled with the impact of propionate on T-cells important in the pathophysiology of autoimmunity. This broad basis supporting the mechanistic action of propionate has been built up over the last 2 decades and supported by studies in the EAE animal model for MS, where disease score is reduced on propionate supplementation. The recent publication of a prospective study showing the benefit of propionate supplementation on MS disease progression suggests this microbial metabolite may have clinical importance in the management of MS and supplementation may be a useful adjunctive tool to current medications.

The potential use of propionate in MS management is grounded in its activity in regulating T-cell profiles and activity. Studies suggest that T-cell modulation is sensitive to the immunological challenge in the body, and this is supported by animal studies showing propionate supplementation is either neutral or beneficial for host immune activity when tackling bacteria and viruses. Studies supporting this claim show outcome data for infections or related inflammatory processes. Whilst the data may show a general beneficial or neutral effect of propionate supplementation on immune activity, the data is of insufficient volume to give a definitive picture of how propionate supplementation can affect the immune system's response to particular pathogens.

However, the nascent data suggest propionate may be useful in the nutritional management of MS (85) and at the same time be neutral or contribute to a normal physiological immune response essential for tackling the pathogenic fungi, bacteria and viruses the body is exposed to.

In conclusion, there is broad mechanistic support for the role of propionate in regulating the immune system *via* modification of T-cell profiles and activity. In the context of auto-immune disease and gut regulation of immunity, propionate and other SCFAs are considered as important mediators of the gut microbiota. In accordance with this, distal outcomes of auto-immune disease such as seen with MS are linked to low levels of propionate due to gut dysbiosis. The use of propionate as a supplemental adjunct to current medical treatment has been strengthened by consistent evidence from animal models (EAE) and a recently published human intervention trial demonstrating long term improvement in disease progression across MS subtypes. Evidence that propionate may also promote T-cell activity in the face of infection further supports that propionate may be a safe nutritional adjunct to MS treatments.

## Author Contributions

DT drafted the manuscript. RV and PC contributed and commented on the manuscript. All authors contributed to the article and approved the submitted version.

## Funding

The funding required for publication is from Dahrt Biocare AS.

## Conflict of Interest

DT and RV have ownership in DAHRT Biocare AS.

The remaining author declares that the research was conducted in the absence of any commercial or financial relationships that could be construed as a potential conflict of interest.

## Publisher’s Note

All claims expressed in this article are solely those of the authors and do not necessarily represent those of their affiliated organizations, or those of the publisher, the editors and the reviewers. Any product that may be evaluated in this article, or claim that may be made by its manufacturer, is not guaranteed or endorsed by the publisher.
